# Lactate‐mediated activation of GPR81 regulates BCR/Abl protein expression in chronic myeloid leukemia cells selected under low oxygen tension

**DOI:** 10.1002/path.6492

**Published:** 2025-10-23

**Authors:** Giulio Menegazzi, Dayana Desideri, Alessio Biagioni, Elisabetta Rovida, Persio Dello Sbarba, Silvia Peppicelli

**Affiliations:** ^1^ Department of Experimental and Clinical Biomedical Sciences Università degli Studi di Firenze Florence Italy

**Keywords:** chronic myeloid leukemia, BCR/Abl, low oxygen, stem cell niche, lactate, GPR81

## Abstract

Chronic myeloid leukemia (CML) is a stem cell‐driven neoplasia characterized by the expression of the constitutively active tyrosine kinase (TK) BCR/Abl. Under low oxygen, a condition that characterizes stem cell niches (SCNs) *in vivo*, the oncogenic BCR/Abl_protein_ is suppressed. Consequently, leukemia stem cells (LSCs) residing within SCNs show resistance to TK inhibitors (TKIs), the first‐line therapy for CML, due to the lack of their molecular target. It is therefore important to deepen understanding of the mechanisms driving BCR/Abl_protein_ suppression to design new strategies able to repress TKI‐resistant LSCs. Our previous studies showed that BCR/Abl_protein_ suppression occurred when glucose approaches completed exhaustion in culture medium. As lactate is the main byproduct of glucose catabolism in low oxygen, in this study we addressed the role of lactate in regulating BCR/Abl_protein_ expression. We found that treatment of CML cells with 2‐DG, which blocks glycolysis and thereby lactate production, or monocarboxylate transporter (MCT) inhibitors, which reduce lactate excretion, enhanced BCR/Abl_protein_ expression and promoted maintenance of a BCR/Abl‐dependent/TKI‐sensitive stem cell phenotype. The effects of MCT inhibition were abolished when exogenous lactate was added to culture medium, resulting in the suppression of BCR/Abl_protein_ expression. Treatment with 3‐hydroxy‐butyrate acid, an antagonist of the GPR81 plasma membrane ‘lactate’ receptor, prevented lactate‐driven BCR/Abl_protein_ suppression, while the selective GPR81 agonist 3‐chloro‐5‐hydroxy‐BA counteracted the maintenance of BCR/Abl_protein_ induced by MCT inhibition. Our results indicate that GPR81 engagement by extracellular lactate determines BCR/Abl_protein_ suppression in low oxygen environments. © 2025 The Author(s). *The Journal of Pathology* published by John Wiley & Sons Ltd on behalf of The Pathological Society of Great Britain and Ireland.

## Introduction

Chronic myeloid leukemia (CML) is a hematopoietic stem cell (HSC) disease characterized by the translocation t(9;22)(q34;q11), resulting in the fusion of the *BCR* and *ABL1* genes. This fusion gives rise to the *BCR‐ABL1* oncogene and consequent production of the constitutively active BCR/Abl tyrosine kinase, resulting in a proliferative advantage of the mutant HSC [1]. The development of tyrosine kinase inhibitors (TKIs) capable of targeting BCR/Abl kinase activity substantially improved CML prognosis, with long‐term control of disease and extended patient survival [2]. However, notwithstanding the excellent response to TKI treatment, some patients still fail to reach complete remission of disease or develop resistance to the treatment, mainly due to the persistence of a TKI‐insensitive leukemia stem cell (LSC) subset, which sustains the so‐called post‐treatment/treatment‐resistant minimal residual disease (MRD) of CML [3, 4, 5]. LSCs, like HSCs, reside within bone marrow (BM) stem cell niches (SCNs), a complex microenvironment characterized by peculiar conditions, such as critical levels of glucose and very low oxygen tension. These features profoundly influence the maintenance of stem cell potential and the sensitivity of LSCs to therapeutic agents [6].

Our previous studies demonstrated that when CML cells were incubated in an atmosphere at very low oxygen tension (0.1%–0.2% O_2_), there is rapid exhaustion of glucose in culture medium (due to the Pasteur effect), paralleled by lactate production and medium acidification [7]. In these conditions, BCR/Abl_protein_ expression and, therefore, BCR/Abl signaling are suppressed in a time‐dependent manner, leading to the selection of a cell subset with stem/progenitor cell potential and BCR/Abl independence that persists *in vitro* and *in vivo* [8, 9, 10, 11, 12]. We later demonstrated that the regulation of BCR/Abl_protein_ expression and stem cell potential in low oxygen environments is related to glucose consumption. Indeed, in the presence of glycolysis inhibitors, or in the absence of glutamine, where glucose consumption slows, the fading‐off of BCR/Abl_protein_ expression and signaling is delayed, so that CML stem cell potential remains TKI‐sensitive [7].

In this study, we investigated the role of lactate in regulating BCR/Abl_protein_ expression and stem cell phenotype in CML cells incubated at very low oxygen tension. The question was whether lactate availability in the glucose‐free zones of SCNs could fuel the maintenance of the BCR/Abl‐independent cell subset, or, on the contrary, act as a surrogate of glucose to prevent BCR/Abl_protein_ suppression, as we found in normoxic conditions [13]. As reported for solid tumors, lactate is exported from glycolytic cells, mainly through the monocarboxylate transporter (MCT)‐4, and accumulates extracellularly where its concentration ranges between 10 and 30 mm [14]. Recent studies have documented elevated lactate concentrations also in the pathological BM microenvironment, particularly in hematologic malignancies, where extracellular lactate levels range from 2 to 8 mm – significantly higher than those observed under physiological conditions, which are typically below 2 mm [15, 16]. Extracellular lactate can be recaptured by other cells, or the glycolytic cells themselves, mainly by means of MCT‐1. Such recapture shapes up a metabolic symbiosis where lactate has been shown to act as a tumor‐promoting metabolic fuel [14]. Alternatively, lactate can behave as an extracellular signaling molecule by binding to the G protein‐coupled receptor 81 (GPR81), also known as hydroxycarboxylic acid receptor 1 (HCAR1) [17]. Recent studies suggest that lactate‐mediated GPR81 activation promotes cancer cell survival and cancer onset and progression [17, 18, 19], as well as metabolic reprogramming [19, 20] and immunosuppression [21, 22].

MCT inhibition, which caused an overall reduction of lactate excretion, maintained BCR/Abl_protein_ expression, whereas the addition of exogenous lactate to the culture medium led to BCR/Abl_protein_ suppression. Thus, extracellular lactate is the key regulator of BCR/Abl_protein_ expression. We found that extracellular lactate downregulated this expression via GPR81 engagement.

## Materials and methods

### Cells and culture conditions

The BCR/Abl‐positive K562 and KCL22 cell lines (WT, TKI‐sensitive), derived from CML patients in blast crisis, were routinely plated at 3 × 10^5^ cells/ml in RPMI 1640 medium supplemented with 10% fetal bovine serum (FBS) (Euroclone, Paington, UK), 2 mm glutamine (Euroclone), 50 U/ml penicillin, and 50 μg/ml streptomycin (Euroclone) and incubated at 37 °C in a water‐saturated atmosphere containing 21% O_2_ and 5% CO_2_. Experiments were performed by incubating cells for 4 days (K562) or 7 days (KCL22) in medium as above at 37 °C, in a water‐saturated atmosphere at very low oxygen tension (0.1% O_2_; 5% CO_2_; 95% N_2_) in a gas‐tight H35 Hypoxic Workstation manipulator/incubator (Don Whitley Scientific, Shipley, Bridgend, UK). The difference in incubation time under low oxygen conditions between K562 and KCL22 cells is attributed to the distinct kinetics of BCR/Abl suppression previously reported [7].

Cell viability was assessed using the trypan blue (Catalogue No.: F‐7378, Sigma‐Aldrich, St. Louis, MO, USA; 0.2 g in 99.8 ml water) exclusion test, counting trypan blue–negative cells with a hemocytometer.

### Reagents

Sodium L‐lactate (Sigma‐Aldrich, Catalogue No.: 867‐56‐1) was dissolved in water and used at different concentrations, from 1 to 10 mm. AXKO (Catalogue No.: HY‐147216; MedChemExpress, Monmouth Junction, NJ, USA), a specific inhibitor of lactate dehydrogenase B, was used at a final concentration of 1 μm; UK‐5099 (Catalogue No.: HY‐15475; MedChemExpress), a potent inhibitor of the mitochondrial pyruvate carrier (MPC), at 10 μm; 3‐chloro‐5‐hydroxybenzoic acid (Catalogue No.: HY‐W016868; MedChemExpress), a GPR‐81 agonist, at 100 μm; 3‐hydroxy‐butyrate (3‐OBA, Catalogue No.: HY‐113378; MedChemExpress), a GPR81 antagonist, at concentrations of 1–10 mm. The glucose analog 2‐deoxy‐d‐glucose (2‐DG) (Catalogue No.: HY‐13966; MedChemExpress) was used at a final concentration of 1 mm. Bindarit (Catalogue No.: HY‐B0498; MedChemExpress), a MCT‐4 inhibitor, was used at 200 μm; AR‐C155858 (Catalogue No.: HY‐13248; MedChemExpress), a selective MCT‐1 inhibitor, at 10 μm; syrosingopine (Catalogue No.: HY‐N4115; MedChemExpress), a MCT‐1/4 inhibitor, at 10 μm. The appropriate concentrations of MCT inhibitors were established using the MTT assay. For selection of the highest nontoxic doses for AR‐C155858 see Silvano *et al* [13]; for bindarit and syrosingopine see supplementary material, Figure S1.

### MTT assay

K562 and KCL22 cells were seeded at 1 × 10^4^ cells per well in 96‐well plates in RPMI 1640 medium without phenol red and incubated at 37 °C in an atmosphere of 5% CO_2_ for 72 h with increasing concentrations of inhibitors. MTT (3‐(4,5‐dimethyl‐2‐thiazolyl)‐2,5‐diphenyl‐2H‐tetrazolium bromide; Catalogue No.: M5655; Sigma Aldrich) was added to the culture medium at a final concentration of 0.5 mg/ml for each well, and the cells were incubated for 2 h. The formazan blue produced was dissolved using dimethyl sulfoxide (DMSO, Sigma Aldrich), and the absorbance was measured at 595 nm using a 550 spectrophotometric microplate reader (Biorad, Milan, Italy).

### Culture repopulation ability (CRA) assay

The CRA assay, an *in vitro* surrogate for the *in vivo* Marrow Repopulation Ability assay, allows us to estimate the stem/progenitor cell potential of cell populations via cell transfer to cultures established in liquid medium rather than cell transplantation into animals. Cells rescued at the end of experiments carried out in atmosphere at low oxygen tension (4 days of incubation for K562, 7 days for KCL22, as previously described in the section ‘*Cells and culture conditions*’) in the absence or the presence of drugs or inhibitors (liquid culture 1: LC1) were washed and replated (3 × 10^4^ cells/ml) into growth‐permissive (21% O_2_, absence of drug) secondary liquid cultures (LC2). The count of the number of viable cells at different times of incubation in LC2 (culture repopulation) gives an estimate of the maintenance of stem cell potential in LC1. The time necessary to reach the peak cell number in LC2, and in particular the presence or absence of a lag phase before repopulation starts, provides information about the TKI resistance or TKI sensitivity, respectively, of stem cell potential.

### Cell lysis and western blotting

Cell pellets were washed twice with ice‐cold PBS, and cell lysis was performed in RIPA buffer supplemented with 100 μm orthovanadate (both from Sigma‐Aldrich) added to the cell pellet. Cells were sonicated for 10 min in an ultrasonic cleaner (VWR, Radnor, PA, USA) and centrifuged for 15 min at 20,000 rcf at 4 °C. Equal amounts of protein in Bolt LDS Sample Buffer (Thermo Fisher Scientific, Segrate, MI, Italy) were separated on Bolt Bis‐Tris Plus gels, 4%–12% precast polyacrylamide gels (Life Technologies, Segrate, MI, Italy). Fractionated proteins were transferred to a PVDF membrane using the iBlot 2 System (Life Technologies). Membranes were blocked for 5 min at room temperature (RT) with the EveryBlot Blocking Buffer (BioRad) and probed overnight at 4 °C with primary antibodies diluted 1:1 in Immobilon® Block – FL (Merck Millipore)/T‐PBS buffer. The primary antibodies used were c‐Abl (K‐12), rabbit polyclonal; phospho‐Crkl (Tyr207), rabbit polyclonal (Cell Signaling Technology, Danvers, MA, USA); Gpr81 (Catalogue No.: 58‐342, ProSci LLC, California, USA), rabbit polyclonal; vinculin (clone hVIN‐1), mouse monoclonal (Sigma‐Aldrich). The anti‐c‐Abl antibody was used to determine BCR/Abl_protein_ expression, given the great differences between the two proteins as for molecular weight and therefore electrophoretic migration. Crkl phosphorylation was used as an indicator of BCR/Abl kinase activity. The membranes were then washed in T‐PBS buffer, incubated for 1 h at RT in 1:1 Immobilon® Block – FL (Merck Millipore)/T‐PBS buffer containing a goat anti‐rabbit IgG Alexa Fluor 750 or a goat anti‐mouse IgG Alexa Fluor 680 secondary antibodies (Invitrogen, Monza, Italy), and protein bands were finally visualized using the Odyssey Infrared Imaging System (LI‐COR, Bioscience).

### Measure of extracellular and intracellular lactate concentrations

For extracellular L‐lactate determination, cells were centrifuged for 5 min at 4 °C, and supernatants were stored at −80 °C until analysis. For intracellular L‐lactate determination, cells were washed with cold PBS, resuspended in 200 ml lactate assay buffer, centrifuged for 5 min at 4 °C at 20,000 rcf, and then the supernatant was collected. Finally, enzymes were removed from the supernatant using the Deproteinizing Sample Preparation Kit (ab204708, Abcam, Prodotti Gianni, Milan, Italy), following the manufacturer's protocol. L‐lactate concentrations were measured using the lactate assay kit (Catalogue No.: ab65330; Abcam), following the manufacturer's protocol, and using a 550 spectrophotometric microplate reader (BioRad) at a wavelength of 570 nm.

### Statistical analyses

Statistical analyses were performed using Student's *t*‐tests or two‐way ANOVA (when more than two samples were compared). Values of *p* ≤ 0.05 were considered statistically significant.

## Results

### Effects of glycolysis inhibition on lactate production, BCR/Abl_protein_ expression, and maintenance of stem cell potential in CML cell cultures incubated at very low oxygen tension

**Figure 1:**
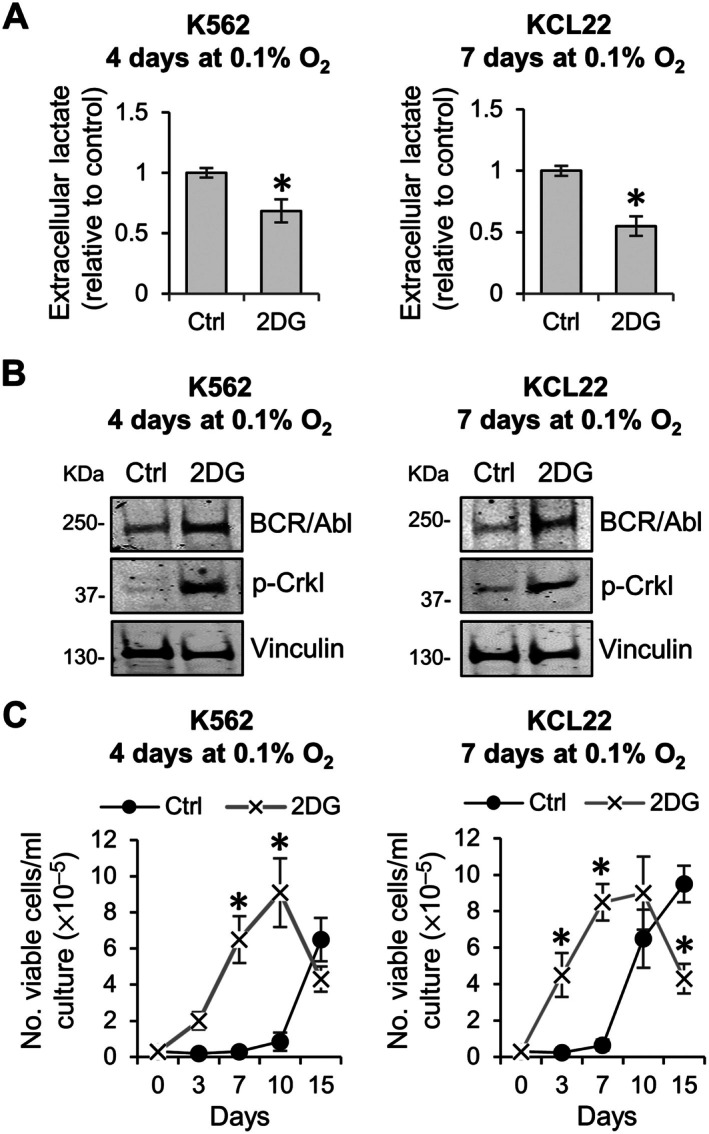
Effects of 2‐DG on BCR/Abl_protein_ expression and signaling and on the maintenance of stem cell potential in low oxygen. K562 or KCL22 cells were seeded at 3 × 10^5^ cells/ml and incubated in atmosphere at 0.1% O_2_ (LC1) for 4 or 7 days, respectively, in the absence or presence of 2‐DG (1 mm). (A) Extracellular lactate concentrations were measured in the culture medium of 2‐DG‐treated cultures and are expressed as a percentage of untreated control. Values are mean ± SD from three independent experiments; *p < 0.05 *versus* control. (B) Total cell lysates were subjected to SDS‐PAGE and immunoblotting with anti‐c‐Abl or anti‐p‐Crkl Ab; anti‐vinculin Ab was used to verify equal protein loading. One representative experiment out of three with similar results is shown. (C) Cells rescued at the end of LC1 were replated at 3 × 10^4^ cells/ml and incubated at 21% O_2_ (LC2). Trypan blue‐negative cells were counted at the incubation times in LC2 as indicated on the *x*‐axis. Values are mean ± SD from three independent experiments; *p < 0.05 *versus* control.

Glycolysis converts one six‐carbon molecule of glucose to two three‐carbon molecules of pyruvate. Under anaerobic conditions, pyruvate cannot be oxidized, and its accumulation is prevented via its conversion to lactate, which is then extruded from cells to avoid cytoplasm acidification, a condition also capable of inhibiting glycolysis. In the experiments shown in Figure 1, to inhibit glycolysis and lactate production, K562 and KCL22 cells incubated in a low oxygen environment for 4 or 7 days, respectively, were treated with the glucose analog 2‐DG, which reduced the amount of lactate released extracellularly (Figure 1A). Moreover, as we previously documented [7], 2‐DG prevented BCR/Abl_protein_ suppression and ensured the maintenance of BCR/Abl signaling, as determined by Crkl phosphorylation (Figure 1B). The cells rescued from day 4 (K562) or day 7 (KCL22) experimental primary liquid cultures (LC1) were then transferred to secondary assay cultures (LC2), incubated at 21% oxygen, to determine the maintenance of stem/progenitor cell potential by measuring LC2 repopulation. Cells of both CML lines rescued from 2‐DG‐treated LC1 exhibited faster repopulation kinetics, characterized by the absence of a lag phase, compared to cells rescued from untreated LC1 (Figure 1C). This pattern was directly correlated with the maintenance of BCR/Abl expression/signaling observed in 2‐DG‐treated cultures. Conversely, CML cells rescued from untreated LC1 repopulated LC2 following a longer lag phase period (Figure 1C), reflecting the kinetics typically observed in cells with suppressed BCR/Abl expression.

### Inhibition of lactate extrusion maintains BCR/Abl_protein_ expression at low oxygen

**Figure 2:**
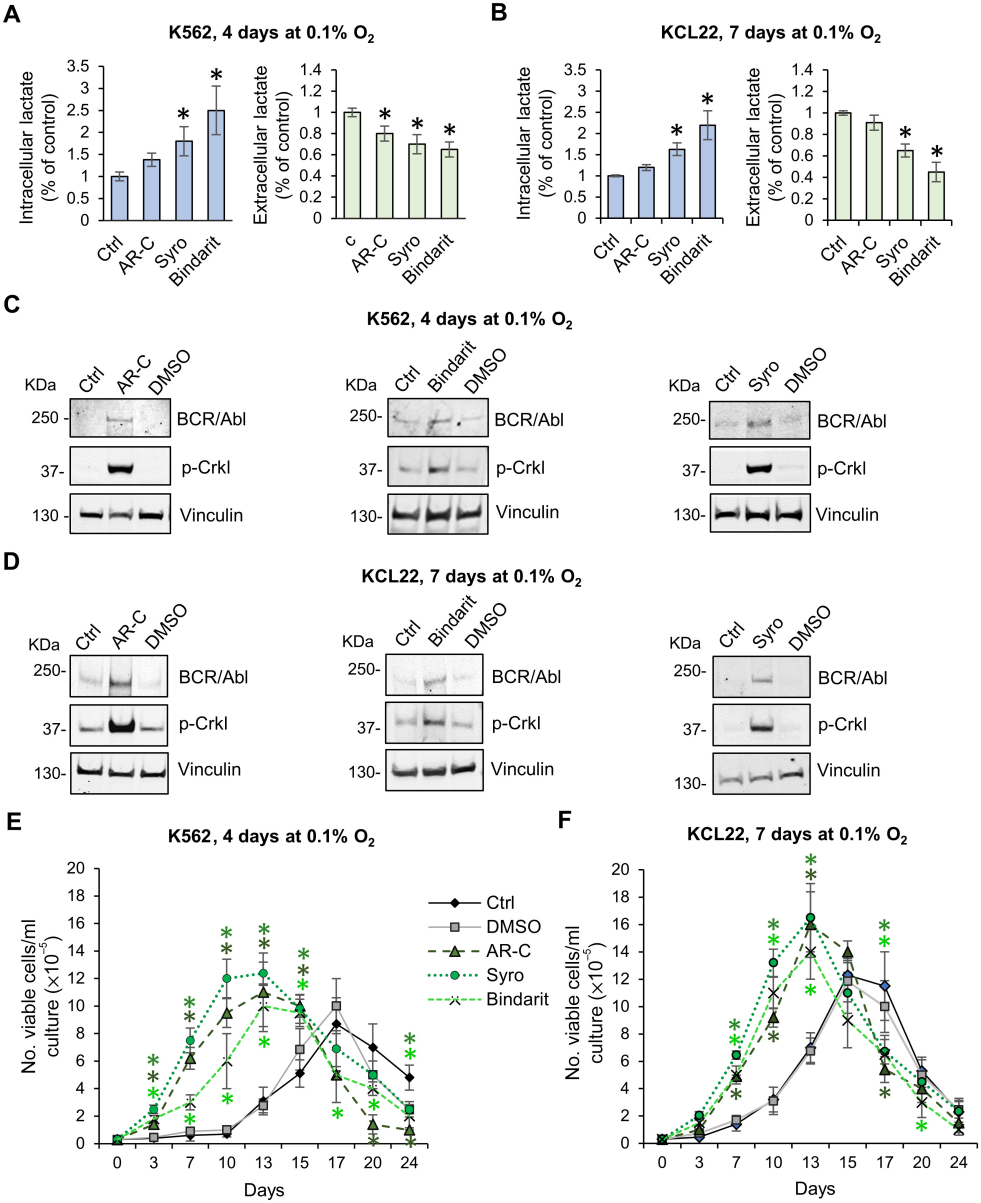
Effects of inhibition of lactate transporters on BCR/Abl_protein_ expression and signaling and on maintenance of stem cell potential in low oxygen. K562 (A, C, E) or KCL22 (B, D, F) cells were incubated at 3 × 10^5^ cells/ml in atmosphere at 0.1% O_2_ (LC1) for 4 or 7 days, respectively, in the absence or presence of lactate transporter inhibitors AR‐C155858 (ARC, 10 μm), bindarit (200 μm), and syrosingopine (Syro, 10 μM), added at time 0 of incubation. (A and B) Intracellular and extracellular lactate concentration expressed as percentage of control. Values are mean ± SD from three independent experiments; *p < 0.05 *versus* control. (C and D) Total cell lysates were subjected to SDS‐PAGE and immunoblotting with anti‐c‐Abl or anti‐p‐Crkl Ab; anti‐vinculin Ab was used to verify the equalization of protein loading. One representative experiment out of three with similar results is shown. (E and F) Cells rescued at end of LC1were replated at 3 × 10^4^ cells/ml and incubated at 21% O_2_ (LC2). Trypan blue‐negative cells were counted at the incubation times in LC2 indicated on *x*‐axis. Values are mean ± SD from three independent experiments; *p < 0.05 *versus* control.

The existence of a cause‐effect relationship between the reduction of extracellular lactate and maintenance of BCR/Abl_protein_ expression in low oxygen, as shown in Figure 1, was addressed by interfering with the transport of lactate through the plasma membrane under conditions where glycolysis was not inhibited (Figure 2). CML cells incubated in low oxygen were treated with several MCT inhibitors: the potent MCT‐1 inhibitor AR‐C155858, the highly selective and noncompetitive MCT‐4 inhibitor bindarit, or the dual MCT‐1/MCT‐4 inhibitor syrosingopine. Cells treated with MCT inhibitors, independently of their specificity for MCT‐1 or MCT‐4, exhibited relatively high intra‐ and low extracellular lactate concentrations (Figure 2A,B). Even though MCT‐1 is primarily known for its role in mediating lactate influx in oxidative cells, and MCT‐4 is generally associated with the export of lactate from glycolytic cells [23, 24], the direction of lactate transport for both carriers depends on the concentration gradients and cellular context. It is therefore possible that, in low oxygen, CML cells, in addition to upregulating MCT‐4 expression via hypoxia‐inducible factor (HIF) stabilization [25], also use MCT‐1 to extrude the large amounts of lactate and H^+^ produced by the high hypoxia‐induced glycolytic rate. Cells treated with any MCT‐1 or MCT‐4 inhibitor showed indeed high intracellular and low extracellular lactate concentrations, although the effect of treatment is much more evident when MCT‐4 is specifically inhibited (bindarit). As in the experiments of Figure 1, cells treated with MCT inhibitors showed high BCR/Abl_protein_ expression and signaling (Figure 2C,D) as well as a BCR/Abl‐dependent stem cell phenotype (Figure 2E,F).

### BCR/Abl_protein_ expression is modulated by extracellular lactate concentration

**Figure 3:**
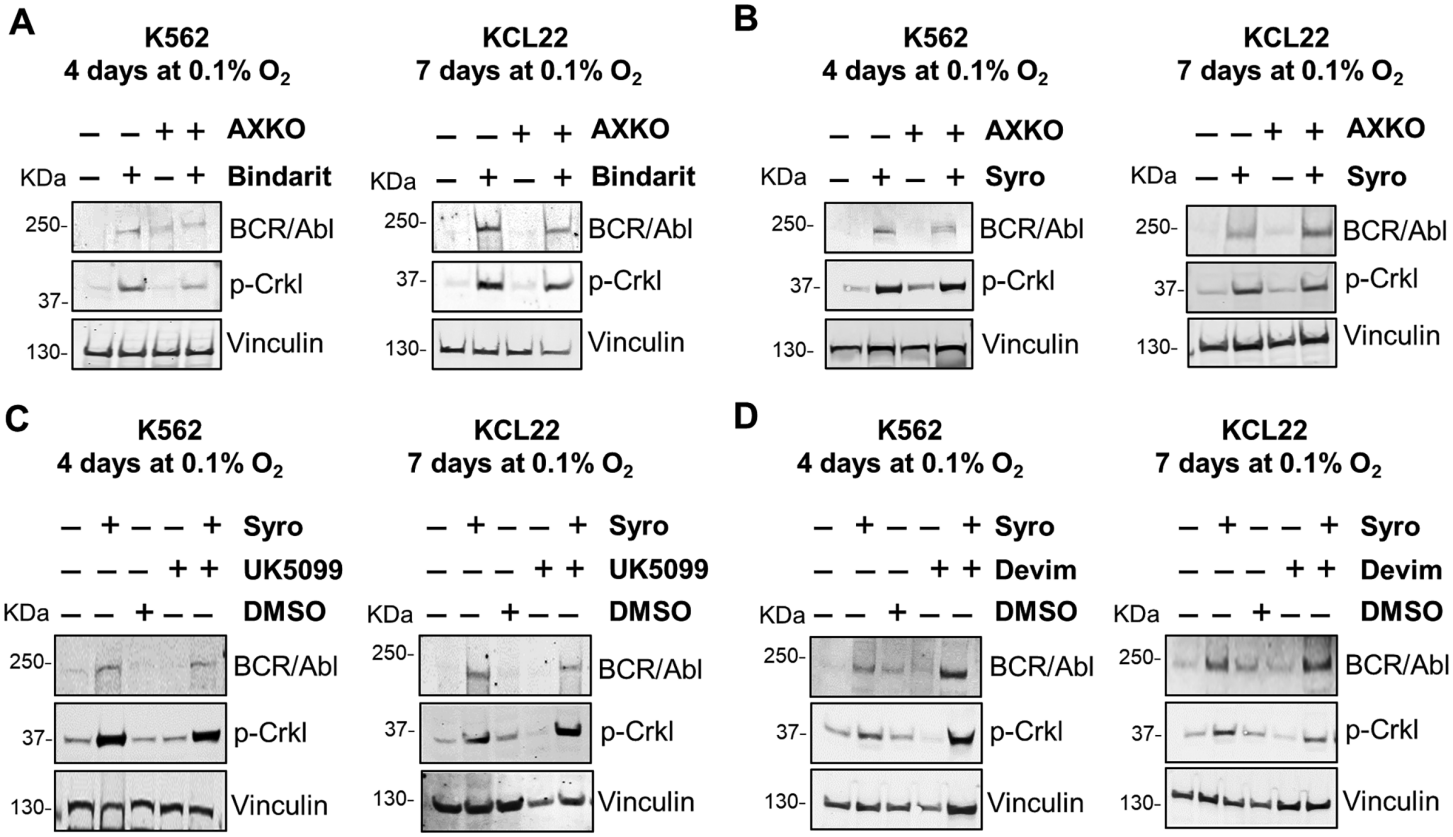
Effects of inhibition of lactate metabolism on BCR/Abl_protein_ expression and signaling in low oxygen. K562 or KCL22 cells were incubated at 3 × 10^5^ cells/ml in a 0.1% O_2_ atmosphere for the indicated times, in the absence or presence of (A) lactate transporter inhibitor bindarit (200 μm) and LDHB inhibitor AXKO (1 μm); (B) lactate transporter inhibitor syrosingopine (10 μm) and LDHB inhibitor AXKO (1 μm); (C) syrosingopine (10 μm) and MPC inhibitor UK5099 (10 μm); (D) syrosingopine (10 μm) and PDH inhibitor devimistat (10 μm). Total cell lysates were subjected to SDS‐PAGE and immunoblotting with anti‐c‐Abl or anti‐p‐Crkl Ab; anti‐vinculin Ab was used to verify equal protein loading. One representative experiment out of three with similar results is shown.

The experiments of Figure 2 demonstrated that lactate traffic through the plasma membrane regulated BCR/Abl_protein_ expression and, in particular, that the maintenance of BCR/Abl_protein_ in low oxygen is associated to high intracellular and low extracellular lactate, in keeping with the results of Figure 1. We therefore tried to determine which of these two parameters was directly linked to the mechanism of BCR/Abl_protein_ modulation. The possible role of high intracellular lactate as carbohydrate fuel in the maintenance of BCR/Abl_protein_ expression was investigated first. The metabolic action of lactate requires its conversion by lactate dehydrogenase (LDH)‐B to pyruvate, which can enter the mitochondrial matrix through the mitochondrial pyruvate carrier (MPC) and be converted by pyruvate dehydrogenase (PDH) to acetyl‐coenzyme A (acetyl‐CoA), able to power the tricarboxylic acid (TCA) cycle and electron transport chain. CML cells incubated in low oxygen were treated with the MCT inhibitors bindarit or syrosingopine, to maintain the intracellular concentration of lactate at high levels, and with the selective LDH‐B inhibitor AXKO‐0046, to inhibit lactate conversion to pyruvate, or the MPC inhibitor UK‐5099, to block the entry of pyruvate into mitochondria, or Devimistat, an agent able to inhibit two key TCA cycle enzymes, PDH and α‐ketoglutarate dehydrogenase. The treatment with either AXKO‐0046 (Figure 3A,B) or UK‐5099 or Devimistat (Figure 3C,D) showed no effect on BCR/Abl_protein_ expression and signaling, the maintenance of which is therefore independent of the catabolism of the abundant intracellular lactate available. That lactate is not used as an energy source in our experimental conditions was expected, since mitochondrial oxidative phosphorylation is inhibited in low oxygen.

**Figure 4:**
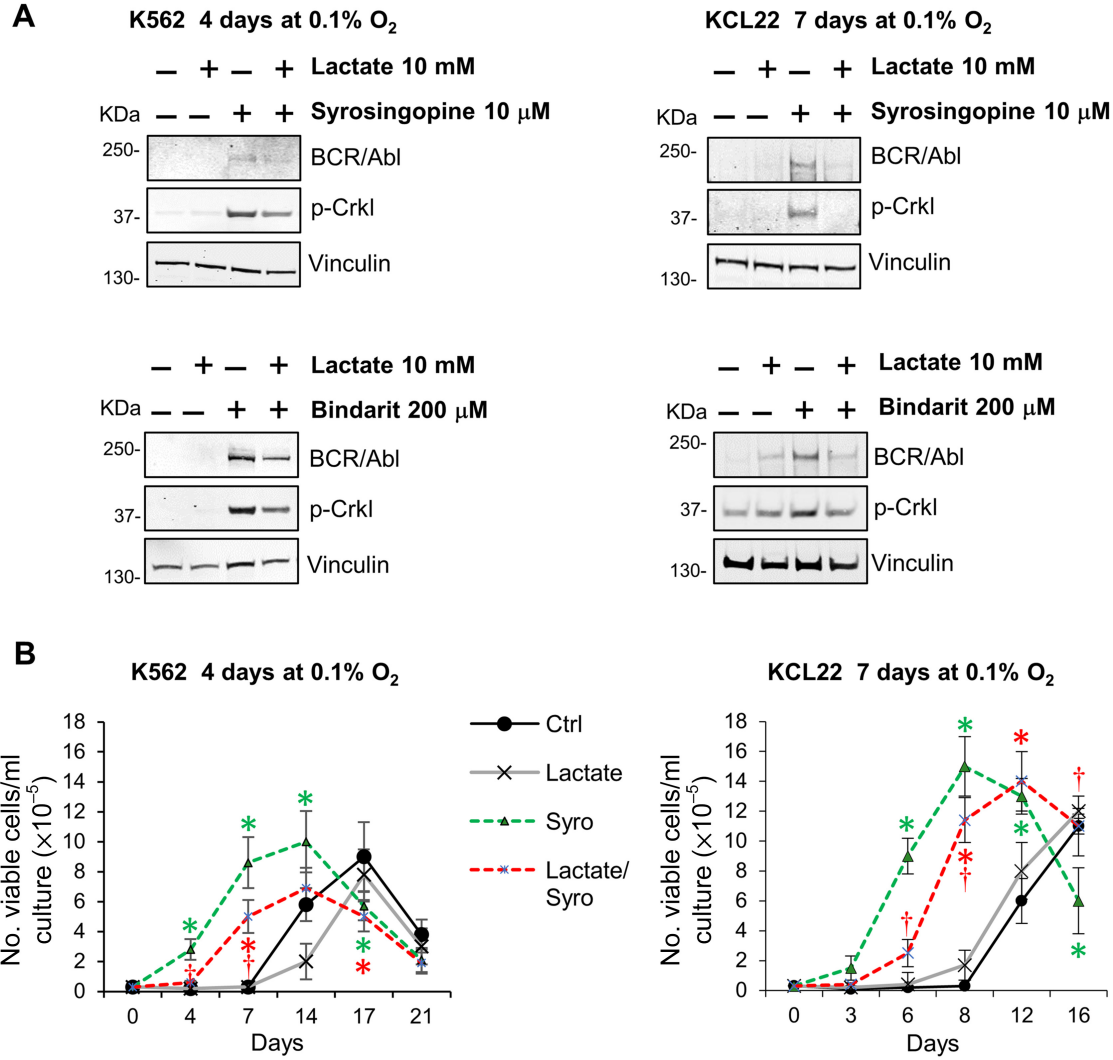
Lactate addition antagonized the effects of lactate transport inhibition on BCR/Abl_protein_ expression and signaling and on the maintenance of stem cell potential in low oxygen. K562 or KCL22 cells were incubated at 3 × 10^5^ cells/ml in atmosphere at 0.1% O_2_ (LC1) for 4 or 7 days, respectively, in the presence or absence of the lactate transporter inhibitors syrosingopine (10 μm) or bindarit (200 μm) and/or lactate (10 mm). (A) Total cell lysates were subjected to SDS‐PAGE and immunoblotting with anti‐c‐Abl or anti‐p‐Crkl Ab; anti‐vinculin Ab was used to verify equal protein loading. One representative experiment out of three with similar results is shown. (B) Cells rescued at the end of LC1 were replated at 3 × 10^4^ cells/ml and incubated at 21% O_2_ (LC2). Trypan blue‐negative cells were counted at the times of incubation in LC2 indicated in abscissa. Values are mean ± SD from three independent experiments; *p < 0.05 *versus* control; ^†^ *p* < 0.05 *versus* syrosingopine.

To evaluate the effects of extracellular lactate on BCR/Abl_protein_ expression, increasing concentrations of lactate (1, 5, 10 mm) were added to CML cultures, where MCT inhibition via syrosingopine treatment determined the maintenance of BCR/Abl_protein_ expression and signaling. Lactate at the highest concentration reduced the maintenance of both BCR/Abl_protein_ expression and signaling consequent to syrosingopine treatment (supplementary material, Figure S2). Such an inhibitory effect of 10 mm lactate on syrosingopine‐ or bindarit‐induced maintenance of BCR/Abl_protein_ expression and signaling (Figure 4A) was paralleled by a relatively slow repopulation of LC2 of CRA assays (Figure 4B), indicating that extracellular lactate was capable of interfering with the maintenance of CML stem cell potential in low oxygen, boosting its BCR/Abl‐independency (LC2 repopulation following a lag phase).

### GPR81 activation drives lactate‐mediated suppression of BCR/Abl_protein_

**Figure 5:**
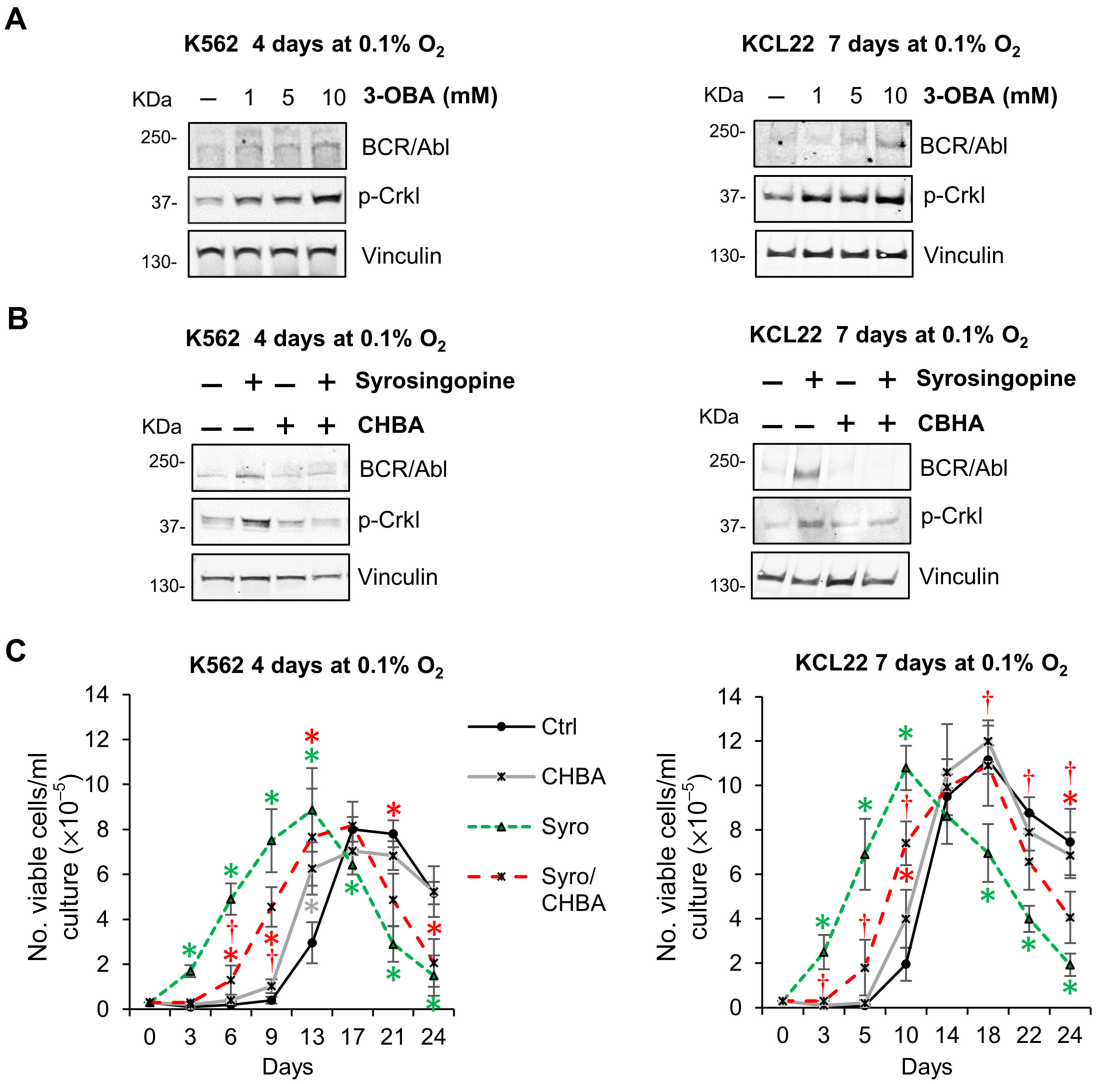
GPR81 activation drives lactate‐mediated suppression of BCR/Abl_protein_ expression and signaling and its effects on the maintenance of stem cell potential in low oxygen. K562 or KCL22 cells were incubated at 3 × 10^5^ cells/ml in atmosphere at 0.1% O_2_ (LC1) for 4 or 7 days, respectively, in the presence or absence of different concentrations of (A) the GPR81 antagonist 3‐OBA (1–10 mm) or (B) the lactate transporter inhibitor syrosingopine (10 μm) and/or the GPR81 agonist 3‐chloro‐5‐hydroxybenzoic acid (GPR81ago). (A and B) Total cell lysates were subjected to SDS‐PAGE and immunoblotting with anti‐c‐Abl or anti‐p‐Crkl Ab; anti‐vinculin Ab was used to verify equal protein loading. One representative experiment out of three with similar results is shown. (C) Cells rescued at end of LC1 were replated at 3 × 10^4^ cells/ml and incubated at 21% O_2_ (LC2). Trypan blue‐negative cells were counted at the times of incubation in LC2 indicated in abscissa. Values are mean ± SD from three independent experiments; *p < 0.05 *versus* control; ^†^ *p* < 0.05 *versus* syrosingopine.

It was recently demonstrated that lactate, in addition to its role as an energy source, can function as a signaling molecule by binding to the plasma membrane GPR81/HCAR1 [26]. We hypothesized that GPR81 activation by extracellular lactate could drive the maintenance of BCR/Abl_protein_‐low CML cells refractory to TKI treatment. First, we verified GPR81 expression in K562 and KCL22 cells and found that long‐term exposure to severe hypoxia (4 days for K562 and 7 days for KCL22) resulted in a downregulation of GPR81 expression (supplementary material, Figure S3A). In contrast, 24‐h exposure induced an increase in GPR81 expression, which was maintained for at least 48 h (supplementary material, Figure S3B). Cell cultures incubated in low oxygen without lactate addition were treated with the GPR81 antagonist 3‐OBA. Our results showed that 3‐OBA prevented BCR/Abl_protein_ suppression at all concentrations used (Figure 5A). In contrast, the treatment with the selective GPR81 agonist CHBA counteracted the MCT inhibitor‐induced maintenance of BCR/Abl_protein_ and signaling (Figure 5B), confirming that GPR81 engagement by lactate inhibits BCR/Abl_protein_ expression. The results of Figure 5B were reflected by the pattern of maintenance of stem cell potential, as CHBA alone did not change the pattern of control cultures significantly, whereas it significantly reduced the effect of MCT inhibition (Figure 5C).

## Discussion

In contrast to aerobic tissue environments, in the low‐oxygen SCN of BM, the mitochondrial function is downregulated, and cells metabolize glucose under anaerobic conditions where glycolysis produces large amounts of lactate, which is then usually exported from cells via MCT‐4. High extracellular lactate concentrations are also typical of zones of solid tumors, where lactate has been shown to sustain cell proliferation, epithelial‐mesenchymal transition, and migration, as well as drug resistance, contributing to cancer progression and recurrence [27, 28]. Here, we are trying to establish a metabolic relationship between SCNs and the core of solid tumors as far as the risk of refractoriness to therapy and relapse of disease is concerned. We indeed characterized the role of lactate in regulating BCR/Abl_protein_ expression and signaling as well as the maintenance of CML stem cell potential in low oxygen.

This study identified extracellular lactate accumulation as a driver of BCR/Abl_protein_ suppression and established that this suppression is subjected to the engagement and activation of GPR81 expressed on the plasma membrane. The scenario shown here is therefore opposite to that we previously outlined in normoxic conditions, where lactate was found to act as a powerful surrogate of glucose to prevent the suppression of BCR/Abl_protein_ expression and signaling and to maintain BCR/Abl‐dependent CML stem/progenitor cell potential [13]. In normoxia, extracellular lactate, once imported into cells by MCT‐1, can indeed represent an alternative energy source. In low oxygen, in contrast, lactate works not as an energy source to sustain the expression of BCR/Abl_protein_ but as a signaling molecule driving or at least contributing to its suppression.

Although MCT‐1 is generally involved in lactate uptake [24], the direction of lactate transport via MCT carriers depends on the concentration gradients of lactate and H^+^. Thus, in low oxygen, MCT‐1 is most probably used prevalently for lactate extrusion, as indicated by the intracellular lactate accumulation determined not only by the treatment with the MCT‐4 inhibitor bindarit but also with the MCT‐1 inhibitor AR‐C155858, in keeping with the results of other studies [29, 30, 31]. The overall block of lactate extrusion caused by MCT inhibition determined a quite remarkable effect, as it prevented BCR/Abl_protein_ suppression. The finding that the BCR/Abl_protein_ expression‐promoting effects of MCT inhibitors can be overridden by the addition of exogenous lactate indicated that it is the extracellular, rather than the intracellular, lactate accumulation that drives BCR/Abl_protein_ suppression.

The role of extracellular lactate has two extremely important implications in our system. The first one, which concerns the course of disease, is that lactate, invariably present in the low‐oxygen microenvironment of SCNs of BM, promotes the selection and maintenance of BCR/Abl‐independent CML stem/progenitor cell potential, which is a crucial factor to determine CML resistance to TKI therapy. This means that lactate enhances the maintenance of MRD and the risk of relapse of disease in CML as much as it stimulates progression, recurrence, and drug resistance of solid tumors. The second implication deals with a more biological issue. Our results indicated that BCR/Abl_protein_ expression and signaling could be maintained (when MCTs are inhibited) in tissue areas at very low oxygen tension. This maintenance requires specific metabolic conditions, such as the presence of lactate or the absence of glutamine [7]. Taken together, these findings indicate that BCR/Abl_protein_ suppression is not mandatory for CML cell adaptation to the harsh SCN microenvironment. As strong BCR/Abl‐generated growth‐promoting signals are most likely unaffordable within the SCN due to energetic constraints [32], one must conclude that the protection of SCN‐adapted cells from the detrimental effects of BCR/Abl‐dependent signals does not necessarily require BCR/Abl_protein_ suppression.

The results relative to extracellular lactate obtained in this study well explain our previous observation that when glucose consumption is slowed down due to the inhibition of glycolysis (2‐DG treatment) or the absence of glutamine, the fading‐off of BCR/Abl_protein_ is delayed [7], whereas when glycolysis is accelerated, as in cell cultures treated with metformin [7], BCR/Abl_protein_ is suppressed more rapidly. These events are probably due to the reduction or increase of lactate production and consequent release into the extracellular microenvironment.

As far as the mechanism of action of extracellular lactate at low oxygen is concerned, since it is not employed as a metabolite and is more active the higher its extracellular concentration, we focused on a possible action of lactate on the external side of plasma membrane. It was recently demonstrated that a specific cell‐surface ‘lactate sensor’, GPR81 [33], once activated by lactate, is crucial for cancer development [34], cell survival [19, 26], angiogenesis [27, 34], chemoresistance [35], and immune resistance [17, 27, 36, 37, 38]. GPR81 has also been shown to be involved in the regulation of cancer cell metabolism [19, 22]. Our study demonstrated that GPR81 was a key factor in the regulation of BCR/Abl_protein_ expression and signaling, as well as in the phenotypical modulation of stem cell potential. Indeed, the treatment with a GPR81 antagonist prevented BCR/Abl_protein_ suppression, while that with a selective GPR81 agonist overrode the MCT inhibitor‐induced BCR/Abl_protein_ maintenance. The downstream connection of GPR81 activation to the mechanism actually regulating BCR/Abl_protein_ expression remains to be elucidated and is the object of current work in our laboratory. We hypothesize that extracellular lactate–mediated activation of GPR81 may suppress BCR/Abl expression through modulation of Yes‐associated protein (YAP). YAP has been shown to enhance the transcriptional activity of c‐Myc [39, 40], a key stem cell marker involved in LSC survival and self‐renewal [41], as well as in the regulation of BCR promoter activity, thereby influencing BCR/Abl expression [42].

## Conclusions

This study demonstrated, for the first time, that a byproduct of energetic metabolism, lactate, is capable of regulating the expression of BCR/Abl oncoprotein in low oxygen. The latter and a high glycolytic rate are features typical of the core of solid tumors as well as of BM and in particular of the zones where SCNs are located. Extracellular lactate, a byproduct of glycolysis in low oxygen environments, was found to drive BCR/Abl_protein_ suppression and thereby to push CML cells endowed with stem cell potential toward a BCR/Abl‐independent phenotype. This phenotype, lacking the TKI target, may limit the effectiveness of BCR/Abl‐targeting therapy. Consequently, lactate present in the BM microenvironment may contribute to the persistence of TKI‐resistant MRD in CML. The upstream mechanism of action of lactate was identified as GPR81 activation. We may speculate that a treatment capable of inhibiting lactate‐mediated GPR81 may antagonize BCR/Abl_protein_ suppression and consequently make leukemic cells homing the SCNs sensitive to TKI action.

## Author contributions statement

GM and SP conceived the study design. PDS acquired funding for the study. GM and DD were responsible for investigation. ER and PDS were responsible for methodology. Data curation and formal analysis were performed by GM and SP. Data visualization was undertaken by GM, AB and SP and AB was responsible for data validation. ER and PDS provided resources, and project administration was conducted by PDS and SP. The study was supervised by SP. GM, PDS and SP drafted the original manuscript. DD and AB reviewed and edited the manuscript. All authors reviewed and approved the final version of the manuscript.

## Supporting information


**Figure S1.** Cell viability analysis performed by MTT assay of K562 or KCL22 cells treated for 72 h with indicated concentrations of syrosingopine (A) or bindarit (B)
**Figure S2**. K562 or KCL22 cells were incubated at 3 × 10^5^ cells/ml in low oxygen 0.1% O_2_ for indicated times in presence or absence of lactate transporter inhibitor syrosingopine (10 μm) and lactate 1, 5, or 10 mm
**Figure S3**. GPR81 expression under normoxic or low oxygen conditions

## Data Availability

The original contributions presented in the study are included in the article; further inquiries can be directed to the corresponding authors.

## References

[1] Jabbour E , Kantarjian H . Chronic myeloid leukemia: 2020 update on diagnosis, therapy and monitoring. Am J Hematol 2020; 95: 691–709. 32239758 10.1002/ajh.25792

[2] Zhou H , Xu R . Leukemia stem cells: the root of chronic myeloid leukemia. Protein Cell 2015; 6: 403–412. 25749979 10.1007/s13238-015-0143-7 PMC4444810

[3] Holyoake TL , Vetrie D . The chronic myeloid leukemia stem cell: stemming the tide of persistence. Blood 2017; 129: 1595–1606. 28159740 10.1182/blood-2016-09-696013

[4] Baykal‐Köse S , Acikgoz E , Yavuz AS , *et al*. Adaptive phenotypic modulations lead to therapy resistance in chronic myeloid leukemia cells. PLoS One 2020; 15: e0229104. 32106243 10.1371/journal.pone.0229104 PMC7046262

[5] Alves R , Gonçalves AC , Rutella S , *et al*. Resistance to tyrosine kinase inhibitors in chronic myeloid leukemia‐from molecular mechanisms to clinical relevance. Cancers (Basel) 2021; 13: 4820. 34638304 10.3390/cancers13194820 PMC8508378

[6] Tabe Y , Konopleva M . Leukemia stem cells microenvironment. In Stem Cell Microenvironments and beyond Advances in Experimental Medicine and Biology (Vol. 1041), Birbrair A (ed). Springer International Publishing: Cham, 2017; 19–32. 10.1007/978-3-319-69194-7_3 29204827

[7] Poteti M , Menegazzi G , Peppicelli S , *et al*. Glutamine availability controls BCR/Abl protein expression and functional phenotype of chronic myeloid leukemia cells endowed with stem/progenitor cell potential. Cancers (Basel) 2021; 13: 4372. 34503182 10.3390/cancers13174372 PMC8430815

[8] Giuntoli S , Rovida E , Barbetti V , *et al*. Hypoxia suppresses BCR/Abl and selects imatinib‐insensitive progenitors within clonal CML populations. Leukemia 2006; 20: 1291–1293. 16710305 10.1038/sj.leu.2404224

[9] Giuntoli S , Tanturli M , Di Gesualdo F , *et al*. Glucose availability in hypoxia regulates the selection of chronic myeloid leukemia progenitor subsets with different resistance to imatinib‐mesylate. Haematologica 2011; 96: 204–212. 21071498 10.3324/haematol.2010.029082 PMC3031687

[10] Cheloni G , Tanturli M , Tusa I , *et al*. Targeting chronic myeloid leukemia stem cells with the hypoxia‐inducible factor inhibitor acriflavine. Blood 2017; 130: 655–665. 28576876 10.1182/blood-2016-10-745588 PMC5942867

[11] Rovida E , Peppicelli S , Bono S , *et al*. The metabolically‐modulated stem cell niche: a dynamic scenario regulating cancer cell phenotype and resistance to therapy. Cell Cycle 2014; 13: 3169–3175. 25485495 10.4161/15384101.2014.964107 PMC4612663

[12] Bono S , Lulli M , D’Agostino VG , *et al*. Different BCR/Abl protein suppression patterns as a converging trait of chronic myeloid leukemia cell adaptation to energy restriction. Oncotarget 2016; 7: 84810–84825. 27852045 10.18632/oncotarget.13319 PMC5356700

[13] Silvano A , Menegazzi G , Peppicelli S , *et al*. Lactate maintains BCR/Abl expression and signaling in chronic myeloid leukemia cells under nutrient restriction. Oncol Res 2022; 29: 33–46. 35131002 10.3727/096504022X16442289212164 PMC9110649

[14] De La Cruz‐López KG , Castro‐Muñoz LJ , Reyes‐Hernández DO , *et al*. Lactate in the regulation of tumor microenvironment and therapeutic approaches. Front Oncol 2019; 9: 1143. 31737570 10.3389/fonc.2019.01143 PMC6839026

[15] Chen Y , Feng Z , Kuang X , *et al*. Increased lactate in AML blasts upregulates TOX expression, leading to exhaustion of CD8+ cytolytic T cells. Am J Cancer Res 2021; 11: 5726–5742. 34873490 PMC8640829

[16] Soto CA , Lesch m , Becker JL , *et al*. Elevated lactate in the AML bone marrow microenvironment polarizes leukemia‐associated macrophages via GPR81 signaling, *bioRxiv* 2023; 2023.11.13.566874. [Not peer reviewed].

[17] Brown TP , Bhattacharjee P , Ramachandran S , *et al*. The lactate receptor GPR81 promotes breast cancer growth via a paracrine mechanism involving antigen‐presenting cells in the tumor microenvironment. Oncogene 2020; 39: 3292–3304. 32071396 10.1038/s41388-020-1216-5

[18] Lundø K , Dmytriyeva O , Spøhr L , *et al*. Lactate receptor GPR81 drives breast cancer growth and invasiveness through regulation of ECM properties and Notch ligand DLL4. BMC Cancer 2023; 23: 1136. 37993804 10.1186/s12885-023-11631-6 PMC10666402

[19] Ishihara S , Hata K , Hirose K , *et al*. The lactate sensor GPR81 regulates glycolysis and tumor growth of breast cancer. Sci Rep 2022; 12: 6261. 35428832 10.1038/s41598-022-10143-w PMC9012857

[20] Longhitano L , Forte S , Orlando L , *et al*. The crosstalk between GPR81/IGFBP6 promotes breast cancer progression by modulating lactate metabolism and oxidative stress. Antioxidants (Basel) 2022; 11: 275. 35204157 10.3390/antiox11020275 PMC8868469

[21] Yang X , Lu Y , Hang J , *et al*. Lactate‐modulated immunosuppression of myeloid‐derived suppressor cells contributes to the radioresistance of pancreatic cancer. Cancer Immunol Res 2020; 8: 1440–1451. 32917658 10.1158/2326-6066.CIR-20-0111

[22] Li X , Chen Y , Wang T , *et al*. GPR81‐mediated reprogramming of glucose metabolism contributes to the immune landscape in breast cancer. Discov Oncol 2023; 14: 140. 37500811 10.1007/s12672-023-00709-z PMC10374510

[23] Dhup S , Kumar Dadhich R , Ettore Porporato P , *et al*. Multiple biological activities of lactic acid in cancer: influences on tumor growth, angiogenesis and metastasis. Curr Pharm Des 2012; 18: 1319–1330. 22360558 10.2174/138161212799504902

[24] Halestrap AP . The SLC16 gene family – structure, role and regulation in health and disease. Mol Aspects Med 2013; 34: 337–349. 23506875 10.1016/j.mam.2012.05.003

[25] Ullah MS , Davies AJ , Halestrap AP . The plasma membrane lactate transporter MCT4, but not MCT1, is up‐regulated by hypoxia through a HIF‐1alpha‐dependent mechanism. J Biol Chem 2006; 281: 9030–9037. 16452478 10.1074/jbc.M511397200

[26] Roland CL , Arumugam T , Deng D , *et al*. Cell surface lactate receptor GPR81 is crucial for cancer cell survival. Cancer Res 2014; 74: 5301–5310. 24928781 10.1158/0008-5472.CAN-14-0319 PMC4167222

[27] Brown TP , Ganapathy V . Lactate/GPR81 signaling and proton motive force in cancer: role in angiogenesis, immune escape, nutrition, and Warburg phenomenon. Pharmacol Ther 2020; 206: 107451. 31836453 10.1016/j.pharmthera.2019.107451

[28] Chen S , Xu Y , Zhuo W , *et al*. The emerging role of lactate in tumor microenvironment and its clinical relevance. Cancer Lett 2024; 590: 216837. 38548215 10.1016/j.canlet.2024.216837

[29] Benjamin D , Robay D , Hindupur SK , *et al*. Dual inhibition of the lactate transporters MCT1 and MCT4 is synthetic lethal with metformin due to NAD+ depletion in cancer cells. Cell Rep 2018; 25: 3047–3058.e4. 30540938 10.1016/j.celrep.2018.11.043 PMC6302548

[30] Grasa L , Chueca E , Arechavaleta S , *et al*. Antitumor effects of lactate transport inhibition on esophageal adenocarcinoma cells. J Physiol Biochem 2023; 79: 147–161. 36342616 10.1007/s13105-022-00931-3 PMC9905156

[31] Saulle E , Spinello I , Quaranta MT , *et al*. Targeting lactate metabolism by inhibiting MCT1 or MCT4 impairs leukemic cell proliferation, induces two different related death‐pathways and increases chemotherapeutic sensitivity of acute myeloid leukemia cells. Front Oncol 2021; 10: 621458. 33614502 10.3389/fonc.2020.621458 PMC7892602

[32] Cheloni G , Poteti M , Bono S , *et al*. The leukemic stem cell niche: adaptation to “hypoxia” versus oncogene addiction. Stem Cells Int 2017; 2017: 4979474. 29118813 10.1155/2017/4979474 PMC5651121

[33] Lee DK , Nguyen T , Lynch KR , *et al*. Discovery and mapping of ten novel G protein‐coupled receptor genes. Gene 2001; 275: 83–91. 11574155 10.1016/s0378-1119(01)00651-5

[34] Lee YJ , Shin KJ , Park S‐A , *et al*. G‐protein‐coupled receptor 81 promotes a malignant phenotype in breast cancer through angiogenic factor secretion. Oncotarget 2016; 7: 70898–70911. 27765922 10.18632/oncotarget.12286 PMC5342597

[35] Wagner W , Kania KD , Blauz A , *et al*. The lactate receptor (HCAR1/GPR81) contributes to doxorubicin chemoresistance via ABCB1 transporter up‐regulation in human cervical cancer HeLa cells. J Physiol Pharmacol 2017; 68: 555–564. 29151072

[36] Su J , Mao X , Wang L , *et al*. Lactate/GPR81 recruits regulatory T cells by modulating CX3CL1 to promote immune resistance in a highly glycolytic gastric cancer. OncoImmunology 2024; 13: 2320951. 38419759 10.1080/2162402X.2024.2320951 PMC10900271

[37] Feng J , Yang H , Zhang Y , *et al*. Tumor cell‐derived lactate induces TAZ‐dependent upregulation of PD‐L1 through GPR81 in human lung cancer cells. Oncogene 2017; 36: 5829–5839. 28604752 10.1038/onc.2017.188

[38] Guo S , Zhou J , Lou P , *et al*. Potentiated effects of lactate receptor GPR81 on immune microenvironment in breast cancer. Mol Carcinog 2023; 62: 1369–1377. 37249360 10.1002/mc.23582

[39] Xiao W , Wang J , Ou C , *et al*. Mutual interaction between YAP and c‐Myc is critical for carcinogenesis in liver cancer. Biochem Biophys Res Commun 2013; 439: 167–172. 23994632 10.1016/j.bbrc.2013.08.071

[40] Nishimoto M , Uranishi K , Asaka MN , *et al*. Transformation of normal cells by aberrant activation of YAP via cMyc with TEAD. Sci Rep 2019; 9: 10933. 31358774 10.1038/s41598-019-47301-6 PMC6662713

[41] Amaya m , Inguva A , Pei S , *et al*. The STAT3‐MYC axis promotes survival of leukemia stem cells by regulating SLC1A5 and oxidative phosphorylation. Blood 2022; 139: 584–596. 34525179 10.1182/blood.2021013201 PMC8796651

[42] Sharma N , Magistroni V , Piazza R , *et al*. BCR/ABL1 and BCR are under the transcriptional control of the MYC oncogene. Mol Cancer 2015; 14: 132. 26179066 10.1186/s12943-015-0407-0 PMC4504180

