# Supplementary material for: Lactate‐mediated activation of GPR81 regulates BCR/Abl protein expression in chronic myeloid leukemia cells selected under low oxygen tension
Source: J Pathol. 2025 Oct 23;267(4):399–409. doi: 10.1002/path.6492 (PMC12596911; doi:10.1002/path.6492)
Supplement: Supplementary file 1 — Figure S1. Cell viability analysis performed by MTT assay of K562 or KCL22 cells treated for 72 h with indicated concentrations of syrosingopine (A) or bindarit (B) Figure S2. K562 or KCL22 cells were incubated at 3 × 10^5^ cells/ml in low oxygen 0.1% O2 for indicated times in presence or absence of lactate transporter inhibitor syrosingopine (10 μm) and lactate 1, 5, or 10 mm Figure S3. GPR81 expression under normoxic or low oxygen conditions [file PATH-267-399-s001.docx]

**Lactate-mediated activation of GPR81 regulates BCR/Abl protein expression in chronic myeloid leukemia cells selected under low oxygen tension**

G Menegazzi *et al. J Pathol* <https://doi.org/10.1002/path.6492>

**Supplementary Figures S1–S3**


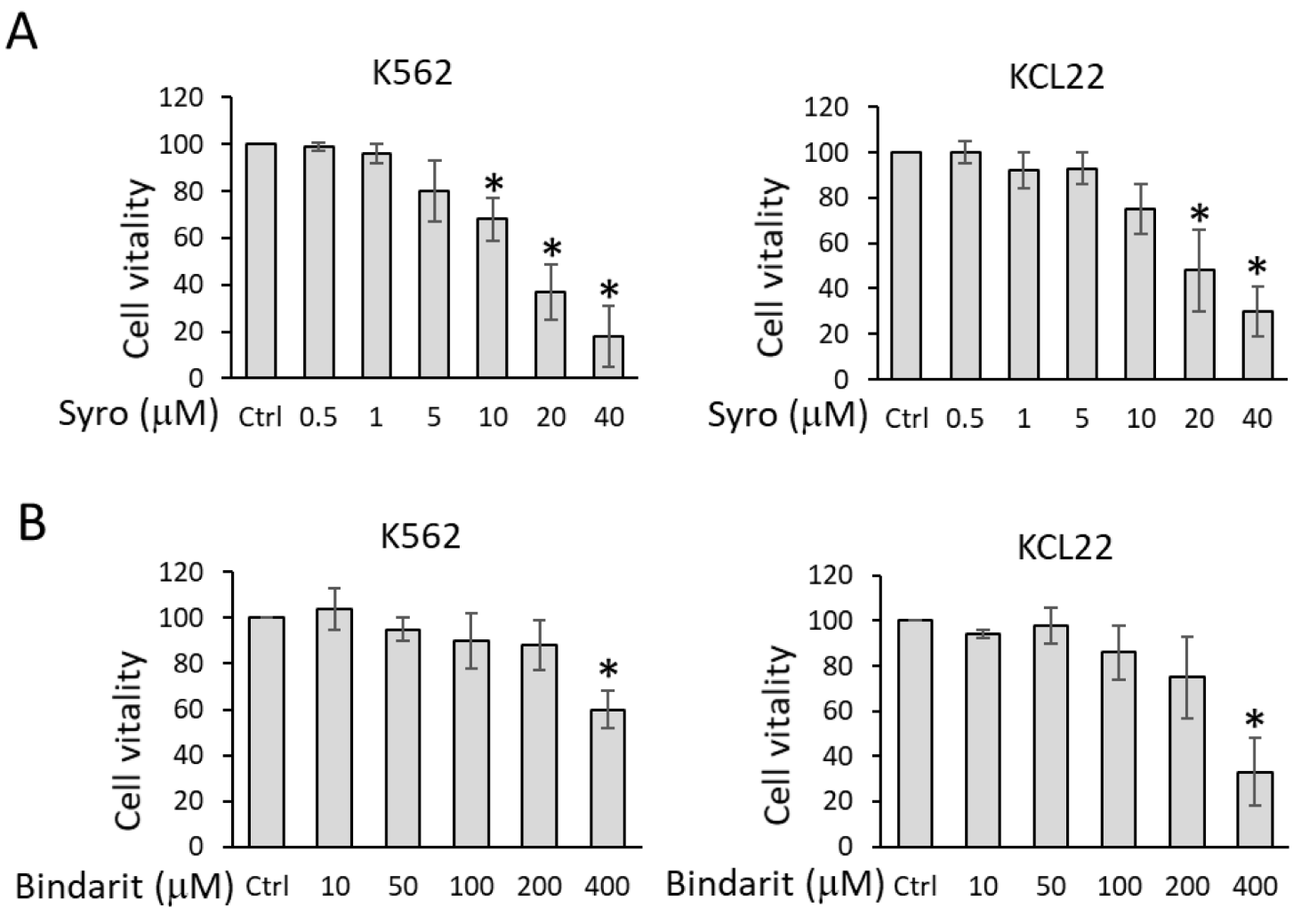


**Figure S1. Cell viability analysis performed by MTT assay using K562 or KCL22 cells treated for 72 h with indicated concentrations of syrosingopine (A) or bindarit (B).** Values are mean ± SD of data obtained from three independent experiments; **p* < 0.05 *versus* control (Ctrl).


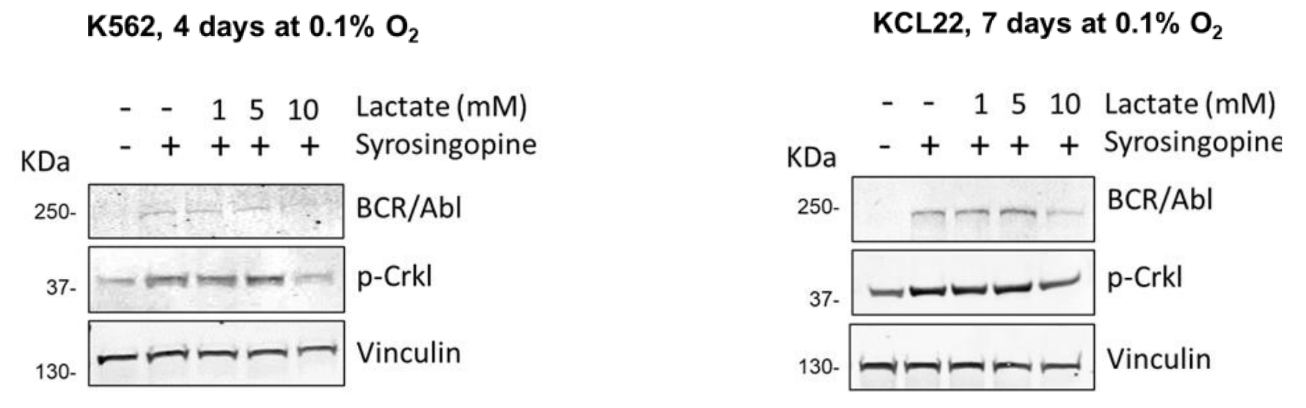


**Figure S2. K562 or KCL22 cells were incubated at 3 × 10^5^ cells/ml in low oxygen 0.1% O_2_ for indicated times in presence or absence of lactate transporter inhibitor syrosingopine (10 μM) and lactate 1, 5, or 10 mM.** Total cell lysates were subjected to SDS PAGE and immunoblotting with anti-Abl or anti-p-Crkl Ab; anti vinculin Ab was used to verify the equalization of protein loading.

**
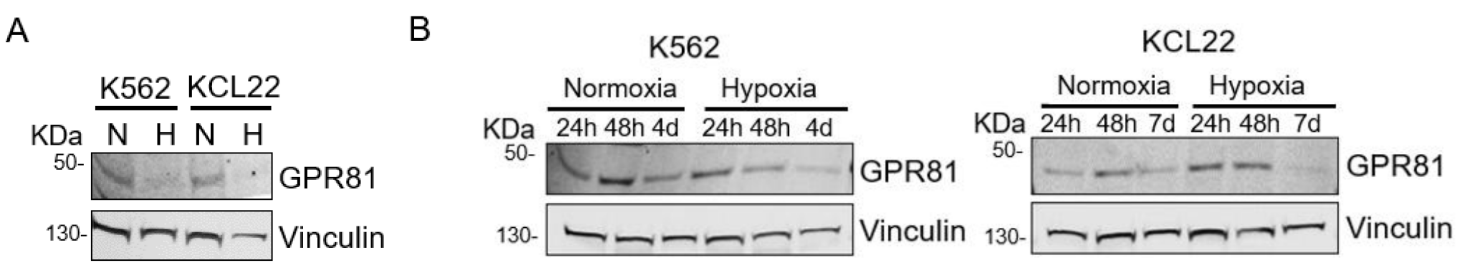
**

**Figure S3**. **GPR81 expression under normoxic or low oxygen conditions**. (A) K562 or KCL22 cells were seeded at 3 × 10^5^ cells/ml under normoxic conditions (N) or at 0.1% O_2_ (H) for 4 or 7 days, respectively. (B) K562 or KCL22 cells were seeded at 3 × 10^5^ cells/ml under normoxic conditions or in an atmosphere at 0.1% O_2_ (hypoxia) for 24 h, 48 h, or 4/7 days. Total cell lysates were subjected to SDS-PAGE and immunoblotting with anti-GPR81; anti-vinculin Ab was used to verify the equalization of protein loading.
